# The application of adjuvant autologous antravesical macrophage cell therapy vs. BCG in non-muscle invasive bladder cancer: a multicenter, randomized trial

**DOI:** 10.1186/1479-5876-8-54

**Published:** 2010-06-08

**Authors:** Maximilian Burger, Nicolas Thiounn, Stefan Denzinger, Jozsef Kondas, Gerard Benoit, Manuel S Chapado, Fernando J Jimenz-Cruz, Laszlo Kisbenedek, Zoltán Szabo, Domján Zsolt, Marc O Grimm, Imre Romics, Joachim W Thüroff, Tamas Kiss, Bertrand Tombal, Manfred Wirth, Marc Munsell, Bonnie Mills, Tung Koh, Jeff Sherman

**Affiliations:** 1 Dept. of Urology, Caritas St. Josef Medical Centre, University of Regensburg, Regensburg, Germany; 2 Dept. d'Urologie, Hopital Necker - Pôle Adulte, Paris, France; 3 Urológiai Sebészeti Osztály, Fővárosi Önkormányzat Péterfy Sándor utcai, Budapest, Hungary; 4 Service Urologie, CHU Kremlin-Bicetre, Kremlin-Bicetre, France; 5 Dept. of Urology, Hospital Universitario Principe de Asturias, Madrid, Spain; 6 Dept. of Urology, Hospital La Fe, Valencia, Spain; 7 Kórház Urológiai Osztály, Fövárosi Önkormányzat Jahn Ferenc Dél-Pesti, Budapest, Hungary; 8 Kórháza Urológiai Osztály, Bács-Kiskun Megyei Önkormányzat, Kecskemét, Hungary; 9 Dept. of Urology, Carl-Gustav Carus University, Dresden, Germany; 10 Dept. of Urology, Semmelweis Egyetem Urológiai Klinika, Budapest, Hungary; 11 Dept. of Urology, Johannes Gutenberg University, Mainz, Germany; 12 Urológiai Osztály, Fővárosi Önkormányzat Bajcsy-Zsilinszky Kórháza, Budapest, Hungary; 13 Urology Unit, Clinique Unversitaire Saint Luc (UCL), Brussels, Belgium; 14 Dept. of Biostatistics, The University of Texas M. D. Anderson Cancer Center Houston, USA; 15 Inspiration Biopharmaceuticals, Laguna Niguel, CA, USA; 16 HorizonTherapeutics, Northbrook, IL, USA

## Abstract

**Introduction:**

While adjuvant immunotherapy with Bacille Calmette Guérin (BCG) is effective in non-muscle-invasive bladder cancer (BC), adverse events (AEs) are considerable. Monocyte-derived activated killer cells (MAK) are discussed as essential in antitumoural immunoresponse, but their application may imply risks. The present trial compared autologous intravesical macrophage cell therapy (BEXIDEM^®^) to BCG in patients after transurethral resection (TURB) of BC.

**Materials and methods:**

This open-label trial included 137 eligible patients with TaG1-3, T1G1-2 plurifocal or unifocal tumours and ≥ 2 occurrences within 24 months and was conducted from June 2004 to March 2007. Median follow-up for patients without recurrence was 12 months. Patients were randomized to BCG or mononuclear cells collected by apheresis after ex vivo cell processing and activation (BEXIDEM). Either arm treatment consisted of 6 weekly instillations and 2 cycles of 3 weekly instillations at months 3 and 6. Toxicity profile (primary endpoint) and prophylactic effects (secondary endpoint) were assessed.

**Results:**

Patient characteristics were evenly distributed. Of 73 treated with BCG and 64 with BEXIDEM, 85% vs. 45% experienced AEs and 26% vs. 14% serious AEs (SAE), respectively (p < 0.001). Recurrence occurred significantly less frequent with BCG than with BEXIDEM (12% vs. 38%; p < 0.001).

**Discussion:**

This initial report of autologous intravesical macrophage cell therapy in BC demonstrates BEXIDEM treatment to be safe. Recurrence rates were significantly lower with BCG however. As the efficacy of BEXIDEM remains uncertain, further data, e.g. marker lesions studies, are warranted.

**Trial registration:**

The trial has been registered in the ISRCTN registry http://isrctn.org under the registration number ISRCTN35881130.

## Introduction

TURB is the therapeutic gold standard for non-muscle invasive BC. Up to 50-70% of cases recur, rendering BC one of the most prevalent malignancies [1]. According to the respective guidelines the use of adjuvant therapy is warranted in patients with intermediate to high risk for tumour recurrence and progression, i.e. multifocal and recurrent disease [1,2]. Two basic forms of adjuvant treatment have been established to date: chemotherapy and BCG. Chemotherapy is antimetabolic and its use recommended in intermediate risk patients [2]. In contrast, BCG stimulates immunoresponse [3]. The use of BCG is suitable for patients with intermediate and high-risk disease and its superiority over chemotherapy has been demonstrated [1,4-6].

While the efficacy of BCG is generally regarded as adequate, its use is debated in low and intermediate risk patients, as its limiting factor is toxicity [1,7,8]. Adverse events (AE) are related to its mode of action, as BCG stimulates immunoreaction and local and systemic inflammatory response occurs. The most frequent immunotherapy linked AEs include constellations of flu- and cystitis-like symptoms. Systemic toxicities, i.e. fever, occur in up to 20% of patients. Due to AEs a considerable portion of patients has been reported to discontinue BCG and many urologists reduce applications [9].

BCG is the most efficacious adjuvant therapy for BC and acts via complex and diverse mechanisms. It is stimulating T-cell mediated local immunoresponse via various cytokines [10,11]. It thus triggers granulocyte related antitumour action [12-15], and macrophage cytotoxicity [11]. BCG has a significant effect on macrophage mobility and phagocytosis. Tumour-infiltrating dendritic cells and tumour associated macrophages counter BCG- effects [16,17]. Natural killer cells and macrophages are viewed as important targets in the cascade of immunoresponse [18,19].

The rationale to apply activated monocytes into the bladder has been studied with regard to the mode of action of BCG [20-22]. Macrophages may be obtained in large quantities by culture of blood monocytes. After activation with interferon-gamma (IFN-γ) ex vivo, macrophages are capable of selectively lysing tumour cells. The antitumoural properties of IFNγ-activated macrophages have been demonstrated in vitro in experimental murine models of human tumours [23]. Monocyte-derived activated killer (MAK) cells are autologous, highly purified, IFNγ-activated macrophages obtained through in vitro culture. Tolerance and preliminary activity of intrapleural infusion of MAK cells have been assessed in mesothelioma [24] and residual peritoneal ovary carcinomas [25].

A prior phase I trial of autologous MAK cells (BEXIDEM^®^) in patients with non-muscle invasive bladder cancer was conducted. Intravesical BEXIDEM therapy was administered after TURB to 17 patients with TaG3 or recurrent TaG2 BC [26]. MAK cells were obtained from autologous mononuclear cells harvested by apheresis and processed by *ex vivo *culture for 7 days and activated with IFN-γ on the last day of culture. The patients received 6 weekly intravesical instillations of approximately 2 × 10^8 ^cells each. Each patient was followed for 1 year or until tumour recurrence, whichever came first. A total of 112 intravesical instillations were performed. No patients discontinued treatment due to an AE and no grade 3 serious AE (SAE) was reported. In 17 patients, 8 tumour recurrences were observed during the 12 months following the first BEXIDEM instillation compared to 34 occurrences despite various adjuvant therapies including BCG in the same patients during the 12 months before (p ≤ 0.0005). Immunoresponse after BEXIDEM was reflected in increased urinary interleukin-8 (IL-8), granulocyte-macrophage colony-stimulating factor (GM CSF), IL-18, elastase and neopterin indicating neutrophil and macrophages activation, respectively [19].

No previous larger data on the use of MAKs exist. The application of viable MAK may trigger various immunological reactions and imply essential systemic risks elusive to smaller series. Thus following the phase I trial, a subsequent larger phase II trial was designed to gather further data on BEXIDEM therapy in patients with non-muscle invasive papillary bladder cancer after TURB. While the secondary objective was to evaluate overall efficacy and recurrence rates in patients treated with BEXIDEM compared to BCG, the primary objective was to demonstrate a superior safety profile of BEXIDEM over BCG.

## Materials and methods

This open-label, randomized study was conducted in 43 centres in Spain, Hungary, France, Germany, Belgium and Luxembourg in accordance with the Declaration of Helsinki, the International Conference on Harmonisation guideline for Good Clinical Practice and local laws between June 2004 and March 2007. The study was sponsored by IDM Pharma, Inc. Ethical oversight was provided by institutional or regional Ethics Committees and signed informed consent was received from each patient. Prior to randomization, patients underwent complete TURB of all suspect lesions. Histopathological examination was conducted according to the 1973 WHO classification and the TNM staging system [27,28]. Patients with plurifocal tumours and patients with a unifocal tumour having a history of at least two occurrences within the prior 24 months were included in the study. Patients were excluded if BC exceeded T1G2, in case of carcinoma in situ, history of tuberculosis, other malignancies within 5 years, active infection and systemic reaction to BCG. Previous BCG treatment was not an exclusion criterion. Patients were randomized to BEXIDEM^® ^or BCG. To minimize prognostic imbalance, patients were stratified according to 3 predefined risk groups (A, B, and C) [29] (table 1). In multiple tumours, the highest grade determined overall tumour grade.

**Table 1:** Stratification according to risk for recurrence prior to randomization according to predefined groups and distribution of BEXIDEM and BCG among the risk groups.

Group	TNM Classification	Number of Tumors	BEXIDEM^®^	BCG
A	Ta Grade 1 T1 Grade 1	Single or multiple Single	26	29
B	Ta Grade 2 T1 Grade 1 T1 Grade 2	Single or multiple Multiple Single	26	26
C	Ta Grade 3 T1 Grade 2	Single or multiple Multiple	23	23

The sample size calculation assumed that 10% and 30% of BEXIDEM and BCG patients, respectively, would experience at least 2 AEs or 1 AE resulting in withdrawal. A sample size of 138 (69 per arm) would provide 80% power to demonstrate this difference with a 2-sided significance level of 0.05.

**Figure 1:**
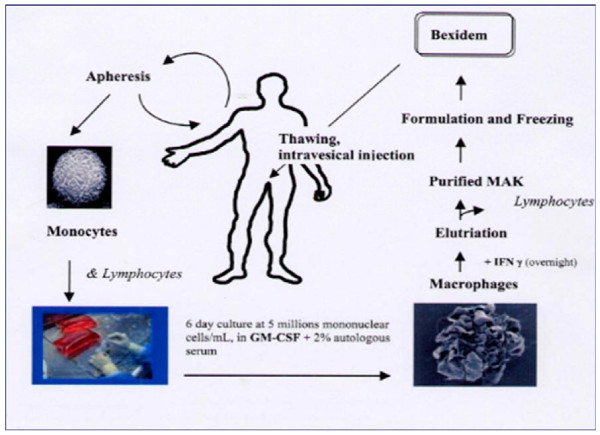
**Scheme of BEXIDEM preparation and administration**.

The BEXIDEM dose and regimen used in the study were based on prior phase I experience [26]. Patients randomized to BEXIDEM had mononuclear cells and plasma collected by apheresis of peripheral blood and shipped to an IDM laboratory in Paris, France. There, monocytes were processed by ex vivo culture in the presence of recombinant human GM-CSF and autologous serum to promote their differentiation to macrophages, which were subsequently activated with IFN-γ. The resulting doses of MAK were cryopreserved, formulated and shipped to the patient's investigational center. Each dose was provided as a frozen sterile, aqueous, suspension of MAK cells containing 10% dimethylsulfoxide (DMSO) and 5% human serum albumin (HSA) in 50 mL cryobags. Each cryobag contained 2 × 10^8 ^MAK cells in a volume of 10 mL. This formulation was kept frozen until use and diluted with HSA prior to administration by bladder instillation (figure 1).

BCG was supplied in packages containing one vial of the freeze-dried product containing 1 to 19.2 × 10^8 ^CFUs. BCG dose and regimen were consistent with the usual adult dose as recommended in the approved product labelling (ImmuCyst, Sanofi Pasteur Limited, Paris, France). Cystoscopy was performed immediately before the first instillation of each treatment cycle to exclude vesical tumour. The first treatment cycle was initiated within 3 to 6 weeks after TURB and consisted of 6 weekly instillations of BEXIDEM or BCG. Maintenance consisted of 2 cycles (at month 3 and month 6) of 3 weekly BEXIDEM or BCG instillations.

Dose reductions of BEXIDEM due to toxicity were not allowed. Dose reductions of BCG to 2/3 or 1/3 of the recommended dose due to toxicity were allowed and had to be documented. Delays in treatment with either BEXIDEM or BCG to allow for resolution of toxicity were allowed.

AEs were assessed prior to every instillation and at 9 weeks, 9 and 12 months after the initial application. Follow-up cystoscopy was performed at 3, 6, 9 and 12 months. The primary safety endpoint was based on the incidence of AEs according to the Common Terminology Criteria for AEs (CTCAE). The relationship of an AE to study treatment was determined by the investigator. AEs were coded using the Medical Dictionary for Regulatory Activities (MedDRA), version 9. Efficacy was evaluated as RFS. All visible lesions detected during follow-up were biopsied and recurrent BC confirmed by histopathology. Progression was defined as recurring BC invading muscle.

Fisher's exact test was used to compare the treatment groups with respect to the proportion of patients who experienced 2 or more treatment related AEs or 1 treatment related AE that resulted in study withdrawal; the distribution of low, normal, and high values for laboratory parameters; and the distribution of normal and abnormal results on physical examination. A 2-sample t-test was used to compare the treatment groups with respect to laboratory values and vital signs at each visit. Fisher's exact test was used to compare the treatment groups with respect to the proportion of patients who experienced tumour recurrence.

## Results

Out of 153 patients randomized, 6 withdrew consent, 2 experienced AEs before treatment, in 1 apheresis was unsuccessful and in 7 protocol violation occurred prior to treatment including other anti-cancer therapy. 137 patients were treated (64 with BEXIDEM, 73 with BCG). All patient characteristics were evenly distributed between the groups (table 2). Previous treatment was most often reported as TURB (45% BEXIDEM, 59% BCG) or TURB plus chemotherapy (23% BEXIDEM, 23% BCG).

**Table 2:** Demographics

Parameter		BEXIDEM N = 75	BCG N = 78
**Age**			
	Mean	63	62.8
	Median	63	64.5
	Range	43-83	27-83
**Sex**			
	Male	62 (83%)	64(82%)
	Female	13 (17%)	14 (18%)
**Ethnic Group**			
	Caucasian	75 (100%)	77 (99%)
	Mediterranean	0 (0%)	1 (1%)

All patients tolerated apheresis and no AEs were reported other than related to peripheral venipuncture. Immunotherapy-related AEs were experienced by 45% (29/64) and 85% (62/73) of BEXIDEM and BCG patients, respectively. The number of patients with either (i) two or more treatment related AEs, or (ii) one treatment related AE resulting in study withdrawal was 31% (20/64) in BEXIDEM and 78% (57/73) in BCG, respectively (p < 0.001). The most common treatment related AEs reported for BEXIDEM patients were hematuria (14%, 9/64), dysuria (13%, 8/64), and urinary tract infection (14%, 9/64), while the most common treatment related AEs reported for BCG-treated patients were dysuria (41%, 30/73), pyrexia (30%, 22/73), pollakisuria (25%, 18/73), and urinary tract infection (38%, 28/73). Serious AEs were experienced by 14% (9/64) of BEXIDEM and 26% (19/73) of BCG treated patients, respectively. Treatment was discontinued due to treatment related AEs in 1 patient treated with BEXIDEM (prostatitis) and 6 patients treated with BCG (table 3). Treatment related AEs reported for patients who discontinued in the BCG group included systemic reaction to BCG with fever, dyspnea, and urinary tract infection.

**Table 3:** Summary of Immunotherapy- related Adverse Events (Patients who received at least one dose of study drug)

Parameter	BEXIDEM^®^ (N = 64)	BCG (N = 73)	P
**No. of patients with AE**	29 (45.3%)	62 (84.9%)	< 0.001
	16 (25.0%)	39 (53.4%)	< 0.001
Grade Moderate			
Grade Severe	7 (10.9%)	12 (16.4%)	0.4593
Relationship Probable	7 (10.9%)	31 (42.5%)	< 0.001
Relationship Definite	3 (4.7%)	26 (35.6%)	< 0.001
			
**No. of patients who discontinued due to an AE**	1 (1.6%)	6 (8.2%)	0.121

The median follow-up for patients without recurrence was 11.9 months (BEXIDEM: 11.6, BCG: 12.2; range 0.1-23.8). Recurrence (with or without progression) occurred in 24/64 (38%) of BEXIDEM and 9/73 (12%) of BCG patients, respectively. Thus recurrence was significantly more frequent in the BEXIDEM arm (p < 0.001). In the BEXIDEM group, 11 of these patients were in risk group A, 11 were in group B, and 4 were in group C. In the BCG group, 6 of these patients were in risk group A, 3 were in group B, and 1 was in group C. Progression to muscle invasive disease occurred in 2/64 (3%) and 1/73 (1%), respectively. One patient in the BEXIDEM group died without relation to treatment or disease.

## Discussion

Non-muscle invasive BC recurs frequently. According to a widely accepted model by Millan-Rodriguez and a more recent model by Sylvester [29,30] patients included into the present study were at intermediate risk for recurrence requiring adjuvant therapy but at low risk for progression according to respective guidelines [1]. BCG is most commonly used in patients at high risk for progression but is also justified in patients at sufficient risk of recurrence as patients included in the present trial. As the efficacy of BEXIDEM could not be reliably judged, no patients at high risk of progression, e.g. CIS, were included in order to avoid undue risks.

While local immunotherapy with BCG is the most effective agent in preventing recurrence, frequent AEs (e.g., cystitis, mild fever) and less common but severe complications (e.g., fever, granulomatous prostatitis) occur [2-4]. The feasibility to develop novel adjuvant agents in addition to chemotherapy or BCG is twofold; for one it would be ideal to combine the efficacy of BCG with a more advantageous AE profile and secondly therapeutic options are limited following failure of one substance [1].

As BCG is an immunotherapeutic and BC is viewed as susceptible to respective targeting, it is feasible to pursue further immunotherapeutical approaches. Autologous MAK cell therapy has been reported as a promising treatment modality including BC [24,17]. While BCG is mediating activation of the immune system via T-cells and its action is not tumour specific, MAK-cells are targeting tumour cells. Their mode of action may be considerably more specific resulting in an improved safety profile [26,19]. However, safety is a concern in treating patients with MAK, as these central agents of immunoresponse could trigger widespread immunological reactions. Hence safety was chosen as the primary endpoint for the present trial and accordingly the protocol defined adverse events in a strict manner, explaining the rather high overall rates of any AEs in both arms (BEXIDEM: 45%; BCG: 85%). Even taking the present and rather strict approach in mind, BEXIDEM appeared safe.

In comparison to BCG, the incidence of SAEs was significantly lower in the BEXIDEM arm, as 14% versus 26% of BEXIDEM and BCG patients experienced serious AEs, and 1 and 6 patients discontinued the protocol due to BEXIDEM and BCG related SAEs, respectively. The safety profile demonstrated in the BCG group was consistent with what would be expected with this approved product and is described in the product labelling and literature [1].

While this phase II trial returned the results expected based on the previous study [26] with respect to its primary objectives, a significantly higher proportion of BEXIDEM versus BCG-treated patients experienced BC recurrence. The agents applied were applied correctly, i.e. BCG in accordance to the current EAU- guidelines and BEXIDEM dosing and regimen in accordance to prior phase I experience [1,26]. Viability of BEXIDEM was routinely assessed and no breech of protocol was noted in processing, handling or administering the product ruling out reduced activity by mishandling.

The efficacy of BEXIDEM is uncertain and three aspects are noteworthy suggesting a careful interpretation of the results. For one, the rather low numbers and events in the prior phase I trial may not reflect the true, i.e. potentially low efficacy of BEXIDEM. Secondly, this phase II trial was underpowered for the secondary clinical end-point and the sample size calculation was apt to reflect safety only in accordance to the primary endpoint. Thirdly the overall numbers of recurrence events were low which has to be attributed to the risk profile of most patients, which in retrospect was inadequately advantageous for assessing efficacy. Fourthly, even if a certain prophylactic effect was present, it may have been overruled by the close to optimal prophylactic effect of BCG. Unfortunately no information on the frequency of previous tumour occurrences was obtained upon inclusion into this trial.

Thus the efficacy of BEXIDEM remains uncertain. Planning the present trial marker lesion studies were extensively discussed but decided against due to concerns that larger tumor burden is a known challenge for immunotherapeutic approaches, which rely on immune cell numbers to overwhelm tumor cell numbers. Further trials are warranted and should adopt the marker lesion concept by observing rather small tumour. Future trials should furthermore include assessment of efficacy by histopathological analysis of bladder tissue biopsies following the application of BEXIDEM immunological panels.

## Conclusions

In this initial randomized trial of autologous MAK cell therapy for non-muscle-invasive BC, BEXIDEM demonstrated an adequate safety profile compared to BCG and no widespread immunological reactions were triggered. Recurrences rates in BEXIDEM were significantly higher compared to BCG. Marker lesions and immunological panels are warranted to assess efficacy of this novel immunotherapeutic agent.

## Abbreviations

AEs: Adverse Events; BCG: Bacille Calmette Guérin; BC: Bladder Cancer; CTCAE: Common Terminology Criteria for AEs; DMSO: Dimethylsulfoxide; GM CSF: Granulocyte-macrophage Colony-stimulating Factor; HAS: Human Serum Albumin; IFN-γ: Interferon-gamma; IL-8: urinary interleukin-8; MAK: Monocyte-derived activated killer cells; MedDRA: Medical Dictionary for Regulatory Activities; RFS: Recurrence free survival; SAE: serious AEs; TURB: transurethral resection of BC.

## Competing interests

The authors declare that they have no competing interests.

## Authors' contributions

MB participated in trial coordination, acquired clinical data, participated in data interpretation and drafted the manuscript; NT, SD, JK, GB, MSC, FJ-C, LK, ZS, DZ, MOG, IR, JWT, TK, BT, MW acquired clinical data and participated in data interpretation; MM performed the statistical analysis; BM, TK and JS participated in trial design and coordination. All authors read and approved the final manuscript.
